# Performance of microscopy and RDTs in the context of a malaria prevalence survey in Angola: a comparison using PCR as the gold standard

**DOI:** 10.1186/1475-2875-12-284

**Published:** 2013-08-13

**Authors:** Cláudia Fançony, Yuri V Sebastião, João E Pires, Dina Gamboa, Susana V Nery

**Affiliations:** 1 CISA Project–Health Research Centre in Angola, Caxito, Angola; 2 Department of Epidemiology & Biostatistics, College of Public Health, University of South Florida, Tampa, FL, USA; 3 Department of Internal Medicine, Hospital Professor Fernando Fonseca, Amadora, Portugal; 4 Clinical Pathology Laboratory of Cova da Beira Hospital, Castelo Branco, Portugal; 5 The University of Queensland, Infectious Disease Epidemiology Unit, School of Population Health, Herston, Queensland, Australia

**Keywords:** PCR, RDT, Microscopy, Malaria, *Plasmodium*

## Abstract

**Background:**

Accurate identification of *Plasmodium* infections in community surveys is essential to successful malaria control. Microscopy and rapid diagnostic tests (RDTs) are the main techniques used to diagnose malaria in field-based surveys. While microscopy is still considered the gold standard, RDTs are growing in popularity as they allow for rapid and inexpensive diagnosis. Using data from a prevalence survey conducted in north-western Angola in 2010, the authors aimed to compare the performance of microscopy and RDTs in identifying *Plasmodium falciparum* infections, using polymerase chain reaction (PCR) as the gold standard.

**Methods:**

Results from 3,307 subjects (1,225 preschool-aged children (zero to five year olds), 1,134 school-aged children (six to 15 year olds) and 948 mothers/caregivers (>15 years of age)), tested for *P. falciparum* infections, were utilized. The sensitivity, specificity, positive, and negative predictive values (PPV and NPV) of microscopy and Paracheck-Pf^®^ were compared using the McNemar’s test and the weighted generalized score Chi-squared test for paired data.

**Results:**

The prevalence of *P. falciparum* infections determined by PCR and microscopy was 15.9% and by Paracheck- Pf^®^ was 16.3%. Compared to microscopy, Paracheck-Pf^®^ had significantly higher sensitivity (72.8% *versus* 60%), specificity (94.3% *versus* 92.5%), PPV (70.7% *versus* 60%) and NPV (94.8% *versus* 92.5%). Both tests had significantly lower sensitivity in mothers (36.8% for microscopy and 43.7% for Paracheck-Pf^®^) than in their children (68.4% in zero to five years-old and 60.6% in six to 15 years-old for microscopy and 80.4% in zero to five year-olds and 76.5% in six to 15 year-olds for Paracheck-Pf^®^).

**Conclusion:**

Both microscopy and RDTs performed suboptimally when compared to PCR. False negativity could be associated with the low parasite density profile of the samples. False positivity may be related to the well-described limitations of those techniques such as level of expertise of microscopists or persistent antigenicity from previous infections in the case of RDTs. Nevertheless, RDTs had enhanced performance comparatively to microscopy in detecting malaria infections, favouring their use in community cross-sectional malaria surveys, where expert performance of microscopy is hard to accomplish.

## Background

Active surveillance through field-based surveys is considered a powerful tool for estimating the burden of malaria, as it identifies sustained foci of transmission perpetuated by asymptomatic carriers and low parasitaemia infections [1-3]. Accurate diagnosis of *Plasmodium* infections is crucial for providing realistic estimates of the burden of malaria and preventing misinformed interventions [4,5].

Microscopy has been the method of choice in determining the prevalence of malaria in epidemiologic surveys, allowing quantification and differentiation of *Plasmodium* species at low cost [6-8]. More recently, rapid diagnostic tests (RDTs) were introduced as screening tools in field-based surveys, as they provide readily available results allowing for treatment *in situ*[6,9,10]. RDTs alone were used to monitor *Plasmodium* infections in malaria surveillance programmes in Tanzania, Gambia, Bangladesh and in the 2006–2007 malaria indicator survey in Angola [11-14].

The choice between microscopy and RDTs is not always clear-cut as the performance of both diagnostic techniques in operational conditions varies depending on transmission intensity, prevalence of infections, and parasite density [5,15-17]. Microscopy is reported to detect about 75% of malaria infections in high transmission areas, whereas in low transmission areas this method has been reported to miss up to 88% of infections [17]. Furthermore, the level of expertise of technicians, quality of the equipment, and workload may lead to inaccurate estimates of parasite density and species differentiation [4,7]. On the other hand, the performance of HRP2-immunochromatography-based RDTs is affected by the detection of persistent antigenicity from previous infections, which leads to false positives and overestimate prevalence [10,18-20]. Moreover, deletions or mutations within the *pfhrp-2* gene and the prozone effect may lead to false negatives [21-25]. Additionally, sensitivity of RDTs can vary due to their vulnerability to extreme temperature and high humidity occurring in field-based surveys [26]. Considering these limitations, the use of RDTs in malaria surveys is only advisable when used in comparison with microscopy, as recommended by WHO and performed in the majority of studies [9,10,18,27-29]. However, reliance on microscopy to measure the performance of RDTs should be approached with caution as this technique itself can be compromised by the limitations described above [1,30-32]. Alternatively, PCR is highly sensitive, detecting low parasitaemia cases missed by other techniques and easily reproducible [33-37]. Nevertheless, it is also highly expensive, time and labour consuming and therefore used in only a few studies for confirmation of prevalence data and to measure the accuracy of microscopy and RDTs [31,38-40].

Using data from a prevalence survey conducted in north-west Angola, this study compared the performance (assessed by sensitivity, specificity, positive, and negative predictive values (PPV and NPV)), of microscopy and Paracheck-Pf^®^ in the detection of *Plasmodium falciparum* infections, using PCR as the gold standard.

## Methods

### Study area

The study was conducted in Bengo province, north-western Angola, in the Health and Demographic Surveillance System (HDSS) study area covered by the CISA project (*Health Research Centre in Angola*, translated) [41]. This is considered a meso-endemic malaria area with stable transmission intensity [42]. The main peak of malaria occurs in the rainy season, between November and May [11]. A recent study from the same geographical area reported that 97% of malaria infections are due to *P. falciparum* but all other human species are present either alone or in mixed infections. Almost 90% of malaria infections are due to *P. falciparum* alone, 6.5% to *P. falciparum* and *P. malariae* together, 3.7% due to *P. ovale curtisi* or *P. ovale wallikeri* alone or in combination with other species and 1.1% due to *P. vivax* alone [43].

### Sample collection

Finger-prick blood samples for microscopy, Paracheck-Pf^®^ and PCR were collected from children and their mothers/caregivers, during a baseline field-based prevalence survey implemented between May and August 2010, as described by Sousa-Figueiredo *et al.*[6]. Of the 3,339 participants initially enrolled in the survey, 3,307 (1,225 preschool-aged children (zero to five year olds), 1,134 school-aged children (six to 15 year olds) and 948 mothers/caregivers (>15 years of age), were tested by the three techniques and included in the present study. No clinical assessment of the participants was done at the time of recruitment.

### Team training

The laboratory diagnosis team was composed of five technicians with pre-university training and previous work experience in public and private health units. A five-day retraining course was provided by the CISA project resident laboratory experts and included theoretical and practical sessions on: finger prick blood collection, thick and thin blood smear preparation, Giemsa staining and slide reading, following standard operational procedures, according to the Basic Laboratory Methods in Medical Parasitology manual from WHO [44]. Field workers and microscopists were also trained on how to perform and read Paracheck-Pf^®^*.* All laboratory technicians were supplied with an operational procedures manual regarding laboratory and field diagnosis.

### Microscopy

Thick and thin blood smears were made on the same slide, air dried and transported to the CISA laboratory where they were stored. The slides were stained with 10% Giemsa for 15 minutes and screened for *P. falciparum* parasites by two independent technicians (double-blind). Discordance in the diagnostic (positivity/negativity) was solved by a third reader and discordance in parasite counts was solved by calculating the mean of the two readings. The agreement between the two laboratory technicians performing the 2 independent readings was 99.9%, Kappa = 0.997, P < 0.001. Assexual parasitaemia was quantified against 200 to 500 leucocytes, assuming a white blood cell count of 8,000/μl as recommended by WHO [45]. A slide was considered negative if no parasite was seen when 500 leucocytes were counted. Quality control readings were performed in randomly selected samples by experienced CISA researchers.

### Paracheck-Pf^®^

The Paracheck-Pf^®^ test was performed accordingly to the manufacturer (Orchid Biomedical Systems, India).

### PCR assay (nested PCR for RNA (SSU-rRNA) amplification)

Blood samples were spotted onto Whatman^®^ 3MMChr filter paper, air dried and stored at 4°C in the CISA laboratory until DNA extraction. Total DNA was extracted using the QIAmp DNA Mini Kit (QIAGEN, UK), following the manufacturer’s instructions. Nested PCR was performed using primers complementary to the *Plasmodium* small subunit ribosomal RNA (SSU-rRNA) gene, as described previously [35,46] and in detail by Fançony [43].

### Statistical analysis

Data were analysed using SAS^®^ software version 9.3. Sensitivity, specificity, positive, and negative predictive values (PPV and NPV) of microscopy and Paracheck-Pf^®^, with PCR as the gold standard, were determined using 2×2 contingency tables and compared using the McNemar’s test (sensitivity and specificity) or the weighted generalized score Chi-squared test (PPV and NPV) for paired data [47]. Exact 95% confidence intervals (CI_95_) were calculated for each measure listed above. For each of the two diagnostic techniques, the Pearson Chi-squared test was used to assess difference in sensitivity, specificity, and predictive values across the three age groups. Statistical significance was set at p < 0.05.

### Ethics approval

Ethical approval was obtained from the Angolan Ministry of Health Ethics Committee. Written informed consent was obtained before inclusion in the study and anti-malarial treatment with ACTs was provided by a nurse or physician when participants had a positive rapid test result. Participants who mentioned feeling unwell were advised to go to the nearest health centre; those deemed with a serious illness were observed by the physician on site if present or transported by the research team to the reference hospital.

## Results

Table 1 provides a matched-sample description of how microscopy and Paracheck-Pf^®^ performed in relation to PCR as the gold standard. In 3,307 samples screened, 525 (15.9%) were identified as positive *P. falciparum* infections by PCR and microscopy and 540 (16.3%) by Paracheck-Pf^®^.

**Table 1:** Matched-sample description of the data

	**PCR positive**			**PCR negative**		
**Microscopy**	**Paracheck-Pf^®^**	**Paracheck-Pf^®^**	**Total**	**Paracheck-Pf^®^**	**Paracheck-Pf^®^**	**Total**
	**Positive**	**Negative**		**Positive**	**Negative**	
Positive	297	18	315	96	114	210
Negative	85	125	210	62	2,510	2,572
Total	382	143	525	158	2,624	2,782

In direct comparison to microscopy, Paracheck-Pf^®^ had significantly higher sensitivity, specificity and predictive values (Table 2). Microscopy correctly identified 315 out of 525 PCR-positive *P. falciparum* infections (60.0% sensitivity, CI_95:_ 55.8-64.2) and 2,572 out of 2,782 PCR-negative samples (92.5% specificity, CI_95:_ 91.4-93.4), with a PPV of 60.0% and NPV of 92.5%. Paracheck-Pf^®^ correctly identified 382 out of 525 PCR-positive *P. falciparum* infections (72.8% sensitivity, CI_95:_ 68.7-76.5) and 2,624 out of 2,782 PCR-negative samples (94.3% specificity, CI_95:_ 93.4-95.2), with a PPV of 70.7% and NPV of 94.8% (Table 2).

**Table 2:** Sensitivity, specificity, predictive values of microscopy and Paracheck- Pf^®^ with PCR as gold standard

		**PCR**	**PCR**	**Sensitivity %**	**Specificity %**	**PPV %**	**NPV %**
		**Positive**	**Negative**	**(CI**_**95**_**)**	**(CI**_**95**_**)**	**(CI**_**95**_**)**	**(CI**_**95**_**)**
		**(n = 525)**	**(n = 2,782)**				
Microscopy	Positive	315	210	60.0	92.5	60.0	92.5
	(n = 525)			(55.8-64.2)	(91.4-93.4)	(55.7-64.2)	(91.4-93.4)
	Negative	210	2,572				
	(n = 2,782)						
Paracheck- Pf^®^	Positive	382	158	72.8	94.3	70.7	94.8
	(n = 540)			(68.7-76.5)	(93.4-95.2)	(66.7-74.6)	(93.9-95.6)
	Negative	143	2,624				
	(n = 2,767)						
			P-value*	<0.0001	<0.0001	<0.0001	<0.0001

*PPV*: positive predictive value; *NPV*: negative predictive value.

CI_95_: 95% confidence interval.

*Obtained from McNemar test (sensitivity, specificity) / Weighted generalized score Chi-square test (PPV, NPV).

Table 3 shows how, for each diagnostic test, the measures of performance varied across the different age groups. The sensitivity of microscopy was lower in mothers (36.8%, CI_95:_ 26.7-47.8) than in their children (68.4%, CI_95:_ 61.9-74.5, in zero to five year-olds and 60.6%, CI_95:_ 53.7-67.2, in six to 15 year-olds) (p < 0.0001). No significant change in the specificity of microscopy was observed across the three age groups. Similarly, the sensitivity of Paracheck-Pf^®^ was lower in mothers (43.7%, CI_95:_ 33.1-54.7) than in their children (80.4%, CI_95:_ 74.7-85.4, in zero to five year-olds and 76.5%, CI_95:_ 70.3-82.1, in six to 15 year-olds) (p < 0.0001) (Table 3).

**Table 3:** Sensitivity, specificity and predictive values of microscopy and Paracheck-Pf^®^ by age group with PCR as gold standard

	**Age**	**Sensitivity %**	**Specificity %**	**PPV %**	**NPV %**
		**(CI**_**95**_**)**	**(CI**_**95**_**)**	**(CI**_**95**_**)**	**(CI**_**95**_**)**
Microscopy	0 - 5	68.4	92.7	67.8	92.9
		(61.9-74.5)	(90.9-94.2)	(61.3-73.9)	(91.1-94.4)
	6 -15	60.6	91.6	62.6	91.0
		(53.7-67.2)	(89.7-93.4)	(55.6-69.3)	(88.9-92.7)
	> 15	36.8	93.0	34.8	93.6
		(26.7-47.8)	(91.1-94.6)	(25.2-45.4)	(91.7-95.1)
P-value*		<0.0001	0.5032	<0.0001	0.0897
Paracheck- Pf^®^	0 - 5	80.4	93.5	73.6	95.5
		(74.7-85.4)	(91.8-95.0)	(67.6-79.0)	(94.0-96.7)
	6 - 15	76.5	93.3	72.4	94.5
		(70.3-82.1)	(91.5-94.8)	(66.1-78.2)	(92.8-95.9)
	> 15	43.7	96.4	55.1	94.4
		(33.1-54.7)	(94.9-97.5)	(42.6-67.1)	(92.7-95.9)
P-value*		<0.0001	0.0064	0.0088	0.4946

Age shown in years; PPV: positive predictive value; NPV: negative predictive value.

CI_95_: 95% confidence interval.

*Obtained from Pearson Chi-squared test.

## Discussion

In this study, the prevalence values determined by microscopy, RDTs and PCR were similar. However, compared to PCR as the gold standard, microscopy and Paracheck-Pf^®^ detected only 60.0 and 72.8% of the true *P. falciparum* infections, respectively. In line with previous studies, parasite density might have determined the low proportion of positive infections detected by microscopy and RDT [17]. As discussed by Sousa-Figueiredo and colleagues, parasite density ranged from moderate to low, decreasing with older age [6]. Accordingly, the present study found that sensitivity of microscopy and RDTs decreased with older age. This is also consistent with the fact that, in malaria-endemic countries, acquired immunity in adult individuals is associated with the presence of submicroscopic infections that are more likely to be undetected by field microscopy or RDTs [5,17,48-50]. On the other hand, false negatives found by Paracheck-Pf^®^ may be explained by deletions or mutations within the *pfhrp-2* gene or by the prozone effect reported by others [21-25,51,52]. Nevertheless, RDTs were significantly more sensitive than microscopy, probably corroborating the ability of RDTs to detect parasites below the threshold of microscopy as previously described [15,53]. Additionally, the 8% false positives detected by microscopy may be explained by erroneous readings performed by the laboratory technicians, mistakenly counting dirt, cell debris and stain artefacts as malaria parasites, whereas the false positives (6%) incorrectly identified by Paracheck-Pf^®^ may be associated with persistent antigenicity from previous infections and with cross reactivity with autoantibodies, non-falciparum malaria and other infectious diseases [5,18,28,30,53-57]. This has resulted in similar specificity between RDTs and microscopy albeit that those from RDTs were significantly higher. Given the results of sensitivity and specificity of microscopy in this study, using it as gold standard for comparison would lead to the misclassification of samples (85 positive and 118 negative) and consequently misleading evaluation of the performance of RDTs. It should be taken into account that even though laboratory technicians who participated in this study were retrained on malaria diagnosis their level of expertise was not formally assessed. Therefore further training and/or a stricter selection of technicians could have increased the performance of microscopy. Despite providing reliable epidemiologic information, the use of PCR is less feasible in studies conducted in developing countries due to the high costs involved [1,40,58]. A pooled PCR, reported to be economically more viable than individual PCR, would provide a feasible alternative for confirming prevalence of infections and evaluating the performance of RDTs or microscopy in determining malaria prevalence [1,58].

In conclusion, given the observed higher sensitivity, specificity and predictive values of RDTs, the data presented here suggest that, for community-based surveys with similar levels of endemicity and transmission rates and where adequate expert performance of microscopy is hard to accomplish, the use of RDTs to determine the prevalence of *P. falciparum* infections is a preferable alternative, if parasite density does not need to be determined. In addition to the low cost and practicability of RDTs, the use of malaria HRP-2/pLDH (pan) combo tests would allow minimizing HRP-2 associated limitations such as false negativity, related to gene deletions or prozone effect; and false positivity associated to persistent antigenicity, extending the feasibility of their use in this context [59]. However both pLDH negative/HRP2 positive and pLDH positive/but HRP2 negative tests would require PCR confirmation in order to: discriminate low parasite density from persistent antigenicity, and confirm the species involved, respectively. When microscopy is the diagnostic technique chosen, thus allowing for determining parasite density, these results suggest that training microscopists and establishing adequate quality control and assurance systems should be set as priorities, in order to guarantee an expert level of microscopy [45]. A systematic review of the publications comparing the performance of different diagnostic techniques in different endemicity and transmission intensity settings is warranted so that informed guidelines regarding the detection and control of *Plasmodium* infections can be developed.

## Competing interests

The authors declare that they have no competing interests.

## Authors’ contributions

SVN was responsible for the study design and coordination and contributed to drafts of the manuscript. CFV and DG carried out the PCR analysis and supervised the microscopy work and field diagnosis. CFV drafted the manuscript. YVS performed the statistical analysis and contributed to drafts of the manuscript. JEP contributed to the statistical analysis and drafts of the manuscript. All authors read and approved the final draft of the manuscript.
